# The Potential of Landscape Plants *Photinia* × *fraseri* and *Pittosporum tobira* as Refuge for Natural Enemies of Pest Insects in Rice–Wheat Rotation Systems

**DOI:** 10.3390/insects17040428

**Published:** 2026-04-16

**Authors:** Qianwen Yang, Qiang Li, Xiaowei Liu, Yajun Yang, Yongming Ruan, Pingyang Zhu, Zhongxian Lu, Chuanwang Cao, Yanhui Lu

**Affiliations:** 1 School of Forestry, Northeast Forestry University, Harbin 150040, China; 13865215415@163.com; 2 State Key Laboratory for Quality and Safety of Agro-Products, Institute of Plant Protection and Microbiology, Zhejiang Academy of Agricultural Sciences, Hangzhou 310021, China; liqiang1120@zju.edu.cn (Q.L.); liuxw@zaas.ac.cn (X.L.); yargiuneyon@163.com (Y.Y.); lvzx@zaas.ac.cn (Z.L.); 3 College of Life Sciences, Zhejiang Normal University, Jinhua 321004, China; ruanym@zjnu.cn

**Keywords:** *Photinia* × *fraseri*, *Pittosporum tobira*, landscape plants, functional plants, predators, parasitoids, rice–wheat system

## Abstract

The rice–wheat rotation is a predominant cropping pattern in China, frequently challenged by pests such as aphids in wheat, and *Chilo suppressalis* and *Cnaphalocrocis medinalis* in rice. This study investigates the potential of two common landscape plants, *Photinia* × *fraseri* and *Pittosporum tobira*, as functional plants for conserving natural enemies across crop cycles. Arthropod communities were systematically monitored using Malaise traps during the wheat, wheat–rice transition, and rice seasons from 2023 to 2024. Results revealed that both species successfully conserved a diverse natural enemy community, though their structural differentiation was strongly driven by seasonal variation, as confirmed by Heatmap and principal component analysis (PCA) (*P.* × *fraseri*: PC1 = 46.3%, PC2 = 23%; *P. tobira*: PC1 = 40.2%, PC2 = 25%). During the wheat season, both plants synergistically supported rich functional guilds, including predatory guilds (e.g., *Episyrphus balteatus*, *Gnathonarium dentatum*, and *Harmonia axyridis*) and parasitic guilds (e.g., *Microplitis tuberculifer* and *Cotesia* spp.). Notably, during the critical wheat-to-rice transition, these shrubs functioned as “habitat anchors,” where *P.* × *fraseri* demonstrated superior retention capacity for functional groups like *Aphidius gifuensis*, mitigating post-harvest habitat fragmentation. During the rice season, distinct functional complementarity emerged: *P.* × *fraseri* appeared to function as a habitat-type plant, potentially providing stable shelter for predatory groups (e.g., spiders and lady beetles), while *P. tobira* appeared to act as a resource-type plant, potentially attracting a significant rebound of parasitoids (e.g., *Xanthopimpla flavolineata*) in August. This mid-summer rebound on *P. tobira* was primarily attributed to its dense evergreen foliage providing a microclimatic refuge, rather than an active flowering resource. Analysis of shared dominant taxa (*H. axyridis*, *Cotesia* spp., and *E. balteatus*) showed highly significant seasonal fluctuations, with peak conservation during the wheat season. This study confirms that *P.* × *fraseri* and *P. tobira* have cross-cycle potential as a “natural enemy bank” in rice–wheat rotation agricultural systems. Their synergistic effects—integrating stable structural shelter with seasonal nutritional subsidies—support the conservation of diverse natural enemy communities throughout the annual crop cycle and significantly enhance the sustained pest control capacity of farmland ecosystems, identifying them as exemplary functional plants for ecological engineering in rice–wheat landscapes and providing a foundation for future studies on biological control efficacy.

## Simple Summary

Modern farming often relies on monocrop fields that lack the natural habitats needed for predators and parasites that suppress pest insects. To address this, we studied whether planting evergreen shrubs like *Photinia* × *fraseri* and *Pittosporum tobira* along the edges of rice and wheat fields could act as a refuge for these natural predators and parasites. By monitoring these shrubs over an entire growing year in Zhejiang Province, China, we found that they support a high variety and number of beneficial insects, especially during critical periods such as harvesting and planting. These shrubs provide a stable home and food sources when the main fields are bare. Our results show that *P.* × *fraseri* is particularly effective during the wheat season, while both plants help maintain a steady population of predatory and parasitic insects throughout the year. Incorporating these flowering shrubs into agricultural landscapes provides foundational support for biological control by maintaining natural enemy populations throughout the year. This approach has the potential to reduce reliance on chemical pesticides, though direct pest suppression efficacy requires further validation through field studies measuring parasitism and predation rates in adjacent crop fields.

## 1. Introduction

With the increasing demand for food, intensive rice–wheat rotation systems have become increasingly prevalent in agricultural production in China and other major rice-producing regions of Asia [1]. Effective pest control is a critical factor for the sustainable development of this cropping model [2]. Currently, chemical control remains the predominant approach to managing key pests such as aphids, rice stem-borers, and rice leaf folders [3]. However, inappropriate pesticide application inevitably leads to pesticide residues exceeding maximum residue limits in harvested grains, diminished crop quality, and food safety risks for consumers [4]. Furthermore, recent studies indicate that excessive pesticide use exerts significant negative impacts on non-target organisms, promotes the development of insecticide resistance in pest insects, and disrupts the inherent balance of agricultural ecosystems [5,6]. Heavy reliance on chemical inputs necessitates a transition toward more sustainable and green pest management strategies [7]. Consequently, achieving green and efficient pest management while safeguarding agricultural production has become a critical challenge for sustainable agricultural development in China [8].

To address this challenge, Ecological Engineering for Pest Management (EEPM) has emerged as an effective and eco-friendly tool for biological control [9]. EEPM is defined as the design and management of agricultural landscapes to enhance the conservation and efficiency of natural enemies, thereby suppressing pest populations through ecological processes [9]. Within this framework, functional plants play a pivotal role [10,11]. Non-crop species are strategically introduced to provide critical resources, such as nectar, pollen, and alternative prey, as well as stable habitats that bridge temporal and spatial gaps in resource availability within simplified landscapes [12].

Functional plants support natural enemies through two primary functional mechanisms: the conservation of predatory guilds and the recruitment of parasitic guilds. First, functional plants enhance the abundance and persistence of predatory natural enemies through habitat stabilization and provision of alternative food sources. Research indicates that *Cnidium monnieri* facilitates the recruitment of ladybugs into wheat fields by releasing specific volatile organic compounds (VOCs) while providing supplementary nectar and dense canopy shelter [13,14]. Similarly, *Cosmos bipinnatus* boosts the reproductive fitness of the predator *Harmonia axyridis* by offering rich pollen and nectar resources [11,15]. Furthermore, woody plants like *Vitex negundo* and *Prunus persica* ensure the year-round persistence of predator populations through phenological complementarity; *V. negundo* provides alternative prey (e.g., leafhoppers) during the wheat–rice transition, and its collective dense structure prevents seasonal population declines during crop fallow periods [16].

Second, these plants play a crucial role in supporting parasitic functional groups by providing essential nutritional subsidies and chemical cues. Adult parasitoids often require sugar sources to maximize their lifespan and fecundity [12]. For instance, the flowers of *Ocimum basilicum* significantly increase the parasitism rates of wasps targeting cornfield pests by providing persistent floral resources [17]. Beyond nutrition, functional plants can actively recruit parasitoids via chemical signaling; for example, *Vigna radiata* releases specific C6 alkene and alcohol volatiles that increase the population of egg parasitoids in corn ecosystems [18]. By providing these multifaceted supports, functional plants act as sustainable biological control agents that conserve and augment diverse natural enemy populations [9,19].

*P.* × *fraseri* and *P. tobira* are evergreen broad-leaved shrubs widely distributed in China [20,21]. The *P.* × *fraseri* is characterized by its rapid growth and perennial, colorful foliage, while *P. tobira* is noted for its high environmental resilience and nectar-rich, prolonged flowering period. Although prior research has extensively documented their capacity for pollutant retention and ornamental use [20,21], their potential as functional plants within agricultural landscapes, particularly as stable habitats during crop transitions, remains under-explored. Specifically, the evergreen canopy of these species may mitigate habitat fragmentation during the critical wheat-to-rice transition period, which is a known bottleneck for natural enemy persistence [22,23]. While specialized functional plants (e.g., flower strips) are effective, their widespread adoption is often hindered by high costs and long establishment periods [24]. By contrast, *P.* × *fraseri* and *P. tobira* offer distinct advantages as they are already integrated into the edges of farmland and rural green belts, requiring no additional costs [20,21]. Based on their biological traits, these landscape plants are hypothesized to serve as alternative functional species. Their dense canopy could provide year-round microclimatic refuges for predatory guilds, while their shoots and floral resources may offer critical nutritional subsidies for parasitoids [23,24,25]. However, empirical evidence confirming these protective effects within rice–wheat rotation systems is currently lacking. It is important to clarify that this study focuses specifically on the potential capacity of conservation to support and maintain natural enemy populations, rather than direct pest suppression outcomes. The latter would require additional measurement of natural enemy dispersal into crop fields and their functional impact on pest populations. To fill this research gap, the present study investigated the capacity of *P.* × *fraseri* and *P. tobira* to conserve natural enemy communities across multiple crop cycles, as a necessary first step toward evaluating their biological control potential.

## 2. Materials and Methods

### 2.1. Materials and Test Sites

The trial was conducted from 2023 to 2024 in a typical rice–wheat rotation area in Deqing County, Huzhou City, Zhejiang Province (120°11′ E, 30°34′ N). Two common landscape species, *P.* × *fraseri* and *P. tobira*, were selected for study. Both species were sourced from landscape plant belts along farmland roads adjacent to intensive agricultural fields. To ensure environmental consistency, all specimens were 3–5 years old and had been transplanted three years prior to the study (circa 2020). These plants were spaced at 5 m intervals, forming a continuous ecological corridor between the farmland roads and the crop matrix. The landscape context consisted of a mosaic of crop fields, drainage ditches, and rural roads. All treatments were managed according to conventional local wheat–rice rotation practices: wheat was sown in early November and harvested in late May, followed by rice transplantation in mid-June and harvest in early November.

### 2.2. Experimental Design and Methods

#### 2.2.1. Experimental Plot Design

The experimental field at the Deqing site consisted of two adjacent but distinct plots to accommodate the different functional hedgerows. The *P.* × *fraseri* plot measured 30 m × 80 m and included an 80 m long uniform hedgerow (Figure S1). The *P. tobira* plot measured 60 m × 80 m and also contained an 80 m long uniformly planted belt (Figure S2). In each hedgerow, three Malaise traps were established as sampling replicates and were randomly placed at different locations with a minimum distance of 20 m between them to ensure independent sampling. All traps were oriented with the opening facing the crop fields to capture arthropod movement between the landscape plants and the crop matrix.

#### 2.2.2. Predator Survey

Natural enemy insects were collected using Malaise traps following the specified sampling regime.

For the *P.* × *fraseri* Treatment Group, sampling in the wheat season was initiated in late April 2024, with collections conducted at 14-day intervals for a total of two sampling events. Sampling was subsequently continued via Malaise traps during the wheat–rice transition period (wheat harvest), starting in June 2024, with a further two sampling events conducted. In the rice season, sampling was launched in August 2024 to coincide with the peak occurrence of rice pests and the stabilization of the arthropod community, and collections were performed every 14 days for a total of six sampling events. For the *P. tobira* Treatment Group, wheat season sampling commenced in May 2024, with collections carried out at 14-day intervals for two sampling events in total. Sampling during the wheat-to-rice transition period (wheat harvest) was also continued using Malaise traps from June 2024, with four sampling events implemented at 14-day intervals, followed by the initiation of rice season sampling in August 2024, where collections were conducted every 14 days for a total of six sampling events (Table S1).

All collected specimens were preserved in 99% ethanol and transported to the laboratory. Predatory natural enemies (e.g., ladybugs and hoverflies) and parasitic natural enemies (primarily parasitoid wasps) were sorted into morphospecies. Taxonomic identification was conducted following the criteria of the Identification and Utilization of Natural Enemies of Rice Pests in China [26], including the full community of landscape plants, not limited to rice pest natural enemies.

### 2.3. Data Analysis

All statistical analyses and data visualizations were performed using R software (v4.3.1). To identify shifts in community structure, Heatmaps were generated to visualize the relative abundance of natural enemy taxa across different seasons. Principal component analysis (PCA) was subsequently conducted to evaluate seasonal clustering patterns and quantify the total variance explained by the primary components (PC1 and PC2). Temporal population dynamics were analyzed by comparing the abundance of natural enemies across the three major agricultural phases. For the shared dominant taxa (*Harmonia axyridis*, *Cotesia* spp., and *Episyrphus balteatus*), one-way analysis of variance (one-way ANOVA) was employed to assess seasonal differences. In cases of heteroscedasticity, the Games–Howell post hoc test was applied to determine significant differences between seasons (*p* < 0.05). Furthermore, to evaluate the synergistic conservation effects, the abundance of shared natural enemies was compared between *P.* × *fraseri* and *P. tobira* within the same growing seasons. All univariate and multivariate statistical tests, along with corresponding figures (including PCA plots, dynamic trend charts, and bar charts), were implemented within the R environment. In all tests, *p* < 0.05 was considered statistically significant and *p* < 0.0001 was considered highly significant.

## 3. Results

### 3.1. Abundance of Insect Species and Taxonomic Distribution Across Photinia × fraseri and Pittosporum tobira

**Figure 1:**
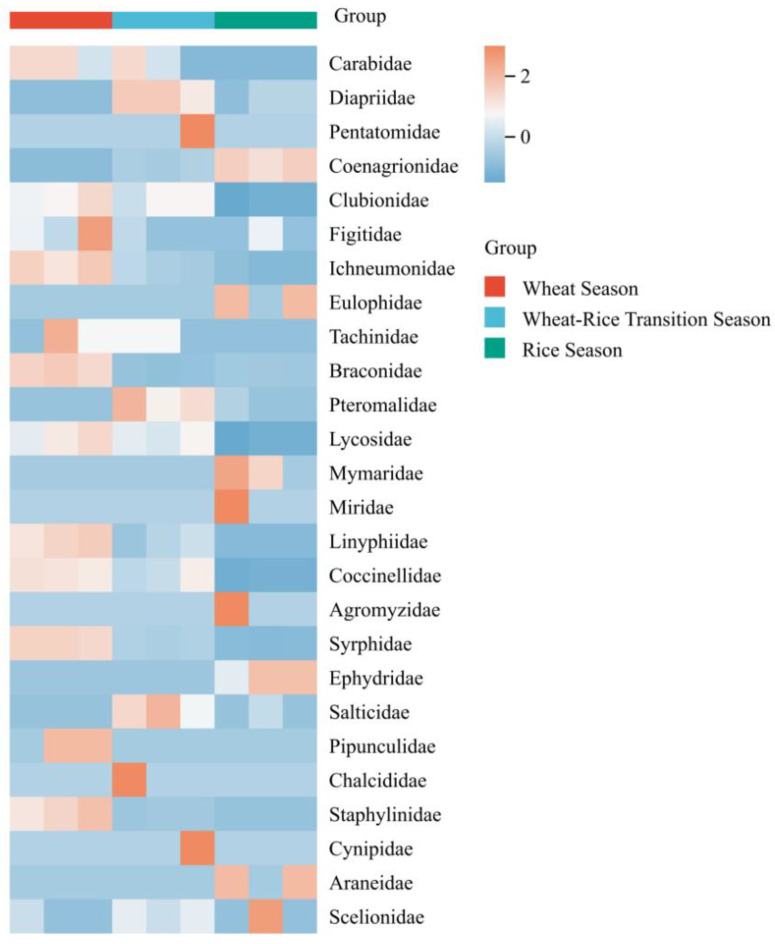
Heatmap of relative abundance of natural enemy taxa associated with *Photinia* × *fraseri* across different planting seasons in 2024. Color scales represent Z-score normalized relative abundance (row-scaled).

**Figure 2:**
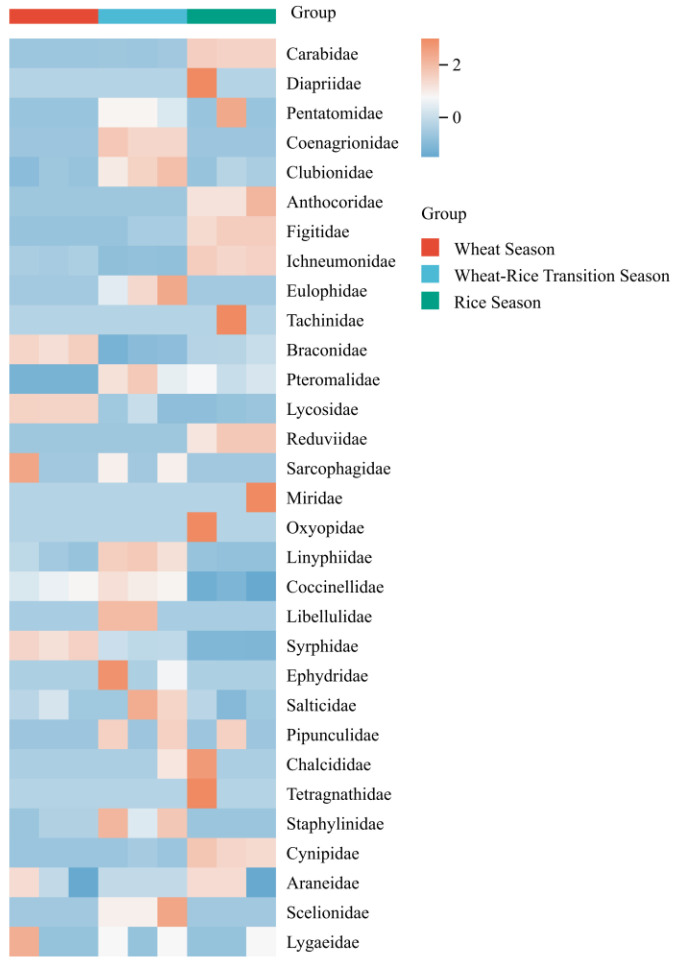
Heatmap of relative abundance of natural enemy taxa associated with *Pittosporum tobira* across different planting seasons in 2024. Color scales represent Z-score normalized relative abundance (row-scaled).

The seasonal dynamics of natural enemy communities associated with landscape plants were elucidated in this study by analyzing the relative abundance and community structure of natural enemy taxa across the wheat season, wheat–rice transition season, and rice season for two agricultural landscape plants: *P.* × *fraseri* and *P. tobira*. Heatmap analysis (Figure 1 and Figure 2) revealed that taxa groups such as Carabidae and Diapriidae associated with *P.* × *fraseri* exhibited higher relative abundance during the wheat season, while the wheat–rice transition season was dominated by taxa groups including Clubionidae and Figitidae. For *P. tobira*, taxa groups such as Anthocoridae and Reduviidae exhibited relatively high abundance in the wheat season, while the wheat–rice transition season was dominated by taxa groups like Braconidae and Pteromalidae.

**Figure 3:**
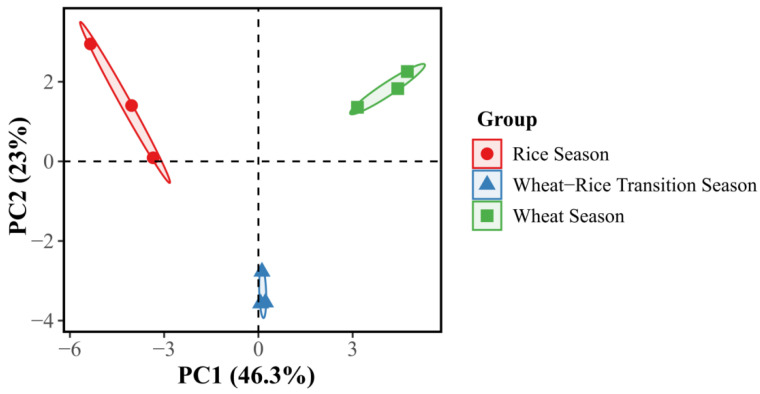
PCA cluster plot of natural enemy taxa associated with *Photinia* × *fraseri* across different planting seasons. Samples exhibit clear clustering by season: red dots represent the rice season, blue triangles represent the wheat–rice transition season, and green squares represent the wheat season, highlighting the differences in natural enemy taxa associated with *Photinia* × *fraseri* among different seasons.

**Figure 4:**
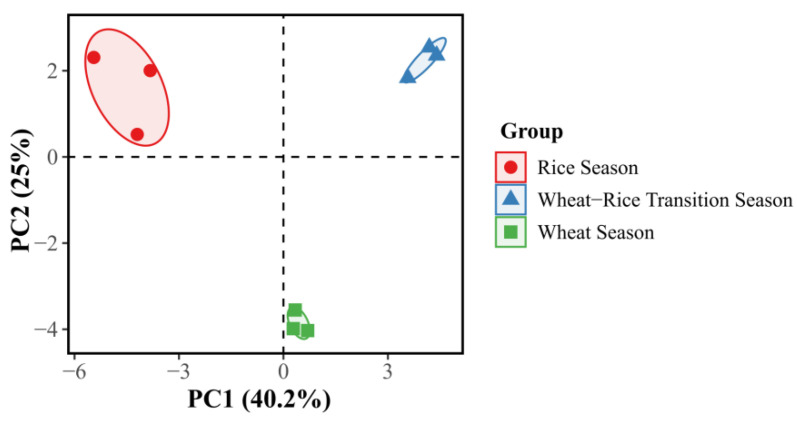
PCA cluster plot of natural enemy taxa associated with *Pittosporum tobira* across different planting seasons. Samples exhibit clear clustering by season: red dots represent the rice season, blue triangles represent the wheat–rice transition season, and green squares represent the wheat season, highlighting the differences in natural enemy taxa associated with *Pittosporum tobira* among different seasons.

Notably, species-specific differences were observed in the composition of dominant natural enemy taxa between the two plant species. Principal component analysis (PCA) (Figure 3 and Figure 4) further demonstrated that principal component 1 (PC1) and principal component 2 (PC2) explained 46.3% and 23% of the total variation in the natural enemy community associated with *P.* × *fraseri*, respectively, while for *P. tobira*, PC1 and PC2 accounted for 40.2% and 25% of the total variation, respectively. Clear seasonal clustering patterns were evident in the natural enemy samples of both plant species, with distinct spatial separation and no overlap among samples from different seasons.

### 3.2. Temporal Dynamics of Insect Abundance on Photinia × fraseri and Pittosporum tobira

The population dynamics of natural enemies associated with both plant species closely align with the crop growing season, peaking during the wheat season. However, subsequent trends diverge markedly. For *P. tobira*, the natural enemy population declines rapidly during the wheat–rice transition period and remains extremely low throughout the subsequent rice season. In contrast, for *P.* × *fraseri*, the population peaks during the wheat season, declines relatively gradually during the transition period, shows a slight rebound in the early rice season, and then declines slowly thereafter.

**Figure 5:**
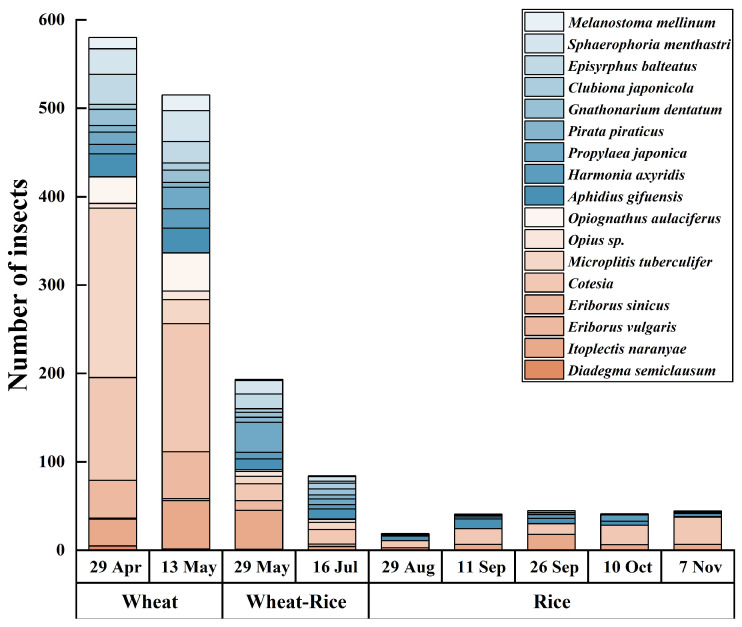
Bar chart showing distribution of dominant natural enemy taxa associated with *Photinia* × *fraseri* across different planting seasons in 2024. Wheat, wheat–rice, and rice represent the wheat season (29 April–13 May), transition season (29 May–16 July), and rice season (29 August–7 November), respectively. The bars represent the total number of individual natural enemies associated with *Photinia* × *fraseri* in different planting seasons. Taxa shaded in blue-toned colors represent predatory natural enemies, and those shaded in orange/peach-toned colors represent parasitic natural enemies (parasitoids).

**Figure 6:**
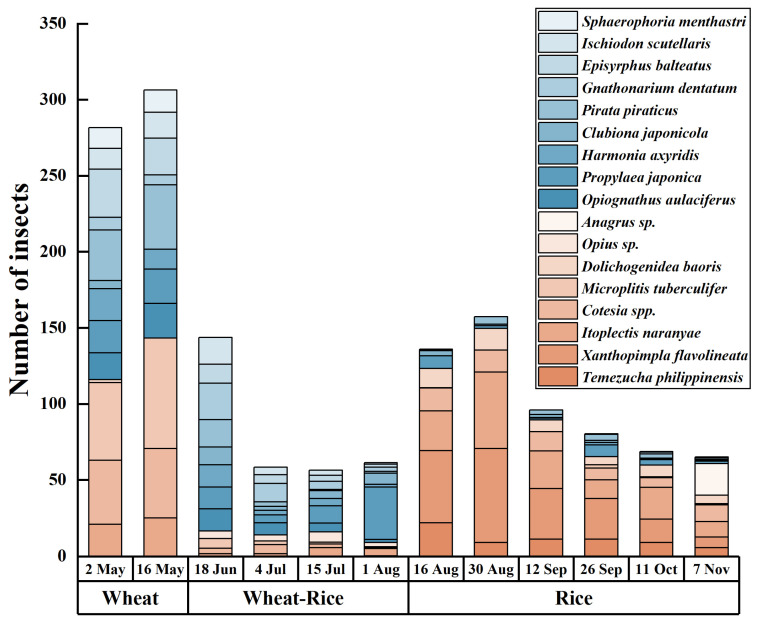
Bar chart showing distribution of dominant natural enemy taxa associated with *Pittosporum tobira* across different planting seasons in 2024. Wheat, wheat–rice, and rice represent the wheat season (2 May–16 May), transition season (18 June–1 August), and rice season (16 August–7 November). The bars represent the total number of individual natural enemies associated with *Pittosporum tobira* in different planting seasons. Taxa shaded in blue-toned colors represent predator natural enemies, and those shaded in orange/peach-toned colors represent parasitic natural enemies (parasitoids).

The wheat season is a critical period for the enrichment of natural enemies on both plant species, with a distinct differentiation in the composition of dominant groups (Table 1 and Table 2). This structural complexity is further supported by alpha diversity analysis (Table 3), which shows that community diversity (Shannon index) and richness (Chao1 and ACE) remain at high levels on both landscape plants during the wheat season, providing a robust biological foundation for pest suppression. On *P.* × *fraseri*, dominant groups primarily comprise parasitoids, including the braconid wasps *Microplitis tuberculifer* and *Cotesia* spp., supplemented by predatory enemies such as *Pirata piraticus*, together accounting for over 60% of the dominant assemblage. On *P. tobira*, the dominant group primarily comprises predatory natural enemies, notably *Gnathonarium dentatum* and *Pirata piraticus*, supplemented by *Harmonia axyridis*, collectively accounting for over 50% of the assemblage. Notably, wolf spiders (*Pirata piraticus*) and ladybird beetles (*Harmonia axyridis*) constitute dominant predatory groups shared across the wheat and rice seasons, demonstrating synergistic conservation effects for common predator communities (Figure 5 and Figure 6).

During the wheat–rice transition period, predator numbers decline markedly for both plant species compared to the wheat season, and interspecific differences become pronounced. Predator numbers on *P. tobira* decline rapidly, with very low populations of *Harmonia axyridis* and *Propylaea japonica*. Although predator numbers on *P.* × *fraseri* also decrease, groups such as *Aphidius gifuensis* remain stable, indicating that this plant has a stronger natural enemy retention capacity than *P. tobira*.

In the rice season, the functional disparity widens further. *P. tobira* shows a significant population rebound in August (Figure 6), primarily driven by parasitic natural enemies such as *Xanthopimpla flavolineata*, although generalist predator numbers remain relatively low. *P.* × *fraseri* maintains a stable abundance of predatory natural enemies (e.g., *Harmonia axyridis* and spiders) during the rice season (Figure 5), demonstrating its more consistent support for predator communities throughout the annual cycle. Subsequently, numbers gradually decline, yet overall natural enemy abundance remains higher than on *P. tobira*, indicating *P.* × *fraseri*’s superior sustained capacity to support natural enemy populations.

### 3.3. Co-Conservation Effects of Photinia × fraseri and Pittosporum tobira on Shared Dominant Natural Enemies

**Figure 7:**
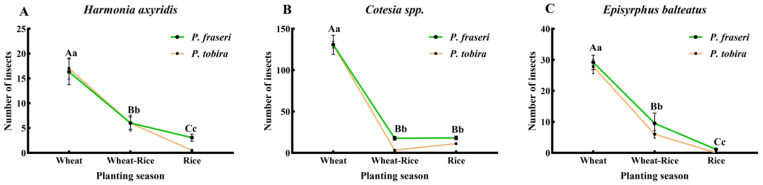
Seasonal population dynamics and significance analysis of shared natural enemy groups on *Photinia* × *fraseri* and *Pittosporum tobira*. (**A**–**C**) The seasonal population changes in *Harmonia axyridis*, *Cotesia* spp., and *Episyrphus balteatus* are shown, respectively. Error bars represent the standard error of the mean (SEM). Wheat, wheat–rice, and rice represent the wheat season, transition season, and rice season. Uppercase letters indicate significant differences among different seasons within *Photinia* × *fraseri*, while lowercase letters indicate significant differences among different seasons within *Pittosporum tobira*. Significance analysis was performed using one-way analysis of variance (one-way ANOVA) followed by Games–Howell’s post hoc test, with the significance level set at *p* < 0.05.

This study further identified three shared dominant natural enemy taxa—*Harmonia axyridis*, *Cotesia* spp., and *Episyrphus balteatus*—with consistent seasonal population trends across *P.* × *fraseri* and *P. tobira* based on the analyses of taxonomic distribution and temporal dynamics of natural enemy communities. Their population variation characteristics and inter-plant synergistic conservation effects are presented below:

The seasonal dynamics of these three shared natural enemies exhibited a highly consistent overall pattern on both plants, with abundance peaking during the wheat season, declining sharply in the wheat–rice transition season, and remaining at the annual lowest level throughout the rice season (Figure 7A–C). One-way ANOVA indicated that seasonal differences in abundance were highly significant for all three taxa on both *P.* × *fraseri* (*F* ≥ 25.52, *df* = 2, 26, *p* < 0.0001) and *P. tobira* (*F* ≥ 42.24, *df* = 2, 35, *p* < 0.0001). Games–Howell multiple comparison tests further revealed that for *H. axyridis* and *E. balteatus*, abundance exhibited a significant and continuous decline from the wheat season and transition period to the rice season (*p* < 0.05). For *Cotesia* spp., abundance in the wheat season was significantly higher than in the transition and rice seasons (*p* < 0.001), while no significant difference was detected between the transition period and the rice season (*p* > 0.05). Within each specific growing season, no significant differences in the abundance of the three shared natural enemies were observed between *P.* × *fraseri* and *P. tobira* (*p* > 0.05). However, we caution that this comparison is based on single plots per species at one location; therefore, the absence of significant differences may reflect limited statistical power to detect treatment effects rather than true ecological equivalence, and the observed patterns may be influenced by site-specific factors.

## 4. Discussion

This study investigated *P.* × *fraseri* and *P. tobira* in southern China, aiming to evaluate their roles in conserving natural enemies within rice–wheat rotation systems and thereby provide novel insights into ecological pest management (EPM) in agricultural landscapes. The results demonstrated that both landscape shrubs exhibit substantial natural enemy conservation potential with apparent functional complementarity: *P.* × *fraseri* is dominated by parasitic natural enemies such as Braconidae, suggesting that it has a “habitat-type” function. This interpretation is supported by its persistent evergreen canopy structure, which provides microclimatic refugia, hypothesized to buffer against physical disturbances. This stable microhabitat acts as a “biological reservoir,” ensuring the survival of sensitive parasitoids during the fallow periods of the surrounding farmland. In contrast, *P. tobira* was dominated by predatory natural enemies such as Anthocoridae, consistent with its “resource-type” function, potentially driven by its nectar-rich floral resources. This functional differentiation is consistent with the core conclusion of previous studies that “plant functional traits determine the composition of natural enemy communities” [9]. Prior research has well documented that evergreen plants and tree phylogenetic diversity tend to support a higher abundance of parasitic natural enemies [22], whereas nectar-producing plants exhibit stronger attractiveness to natural predatory enemies [27,28,29]. The present study extends the current body of knowledge by elucidating the conservation characteristics of functional shrubs in agricultural landscapes, thereby expanding the pool of candidate functional plants for the configuration of vegetation along farmland edges and providing theoretical and practical implications for the construction of diversified natural enemy conservation systems. Seasonal variation is the primary driver regulating the dynamics of natural enemy communities. Principal component analysis (PCA) revealed distinct, non-overlapping seasonal clusters of natural enemy assemblages, with the first two principal components (PC1 and PC2) collectively explaining approximately 65–70% of the total variance. This finding is consistent with previous observations that “the population dynamics of natural enemies are highly synchronized with crop phenological stages” [9,30,31,32]. Notably, *P.* × *fraseri* maintained stable populations of parasitic natural enemies such as *Aphidius gifuensis* during the rice–wheat transition period, whereas *P. tobira* experienced a drastic decline in natural enemy abundance during this period. This differential retention pattern is consistent with the hypothesis that “resource type mediates the retention duration of natural enemies” [33,34,35], indicating that the combination of functional plants with divergent phenological traits could facilitate the year-round stable conservation of natural enemy populations [36,37]. A total of 31 genera of natural enemy insects were collected in this study. The natural enemy communities associated with the two plant species exhibited both specificity and overlap, which partially reflects the mechanism of the ecological “insurance hypothesis” [38]. This is further substantiated by the alpha diversity analysis (Table 3), which shows that taxonomic richness and evenness (e.g., Shannon and Chao1 indices) remain significantly high during the wheat and transition seasons. This high level of functional redundancy within the shrub-associated community may provide a buffer against environmental perturbations, such as crop harvesting, thereby maintaining the continuity of pest control services in the landscape [39,40,41]. In addition to community stability, a critical finding of this study is the highly consistent seasonal dynamics of three dominant shared natural enemies between the two plant species: *Harmonia axyridis*, *Cotesia spp.*, and *Episyrphus balteatus*. Specifically, their populations reached peak abundance during the wheat season, decreased sharply during the rice–wheat transition period, and remained at the lowest levels during the rice season. Additionally, no significant differences in population abundance of these natural enemies were detected between the two plant species during the same season (*p* > 0.05). The peak abundance of natural enemies during the wheat season was primarily driven by the enrichment of food resources and high habitat suitability. During the wheat season, pest populations such as aphids in wheat fields reach their annual peak, providing abundant prey or host resources for dominant natural enemies. Simultaneously, the emerging tender shoots of *P.* × *fraseri* supplied alternative prey, while the flowering period of *P. tobira* offered pollen and nectar for nutritional supplementation. The integration of these resources from landscape shrubs with pest resources in agricultural fields promoted the peak growth of natural enemy populations [42], which aligns with the mechanism proposed by previous studies that “food resource availability is a key driver of natural enemy population dynamics” [43,44]. Furthermore, resource saturation in the agricultural ecosystem reduced the distribution differences in natural enemies between the two plant species, consistent with the widely recognized view that “resource saturation attenuates the niche differentiation of natural enemies among functional plants” [45].

The core cause of the sharp decline in natural enemy populations during the rice–wheat transition period is ecological disturbance and resource disruption in farmland. Wheat harvesting and rice transplanting lead to drastic changes in vegetation structure, resulting in a sharp reduction in pest resources. Agricultural practices such as ploughing destroy habitats, forcing natural enemies to re-distribute between crop fields and the surrounding semi-natural habitats. This aligns with previous research findings that “agricultural operations and other ecological disturbances can significantly reduce natural enemy populations” [46,47,48]. Concurrently, the supply of both plant resources reaches a low point: *P. tobira* pollen and nectar become depleted, while the availability of *P.* × *fraseri* as alternative prey diminishes. This mismatch between resource supply and natural enemy demand determines conservation effectiveness [49,50]. During this phase, large-scale ecological disturbances dominate natural enemy dynamics, while the influence of plant microhabitat variation weakens. Natural enemy abundance remained at its annual low during the rice season, primarily due to mismatched ecological requirements with the wetland habitat of paddy fields [51]. With insufficient plant resources, dominant natural enemies such as *Harmonia axyridis*, which are better adapted to terrestrial environments, find the humidity and vegetation conditions unsuitable for habitation and reproduction. This aligns with prior research on terrestrial natural enemy adaptation in paddy fields [52]. Previous studies have confirmed that wetland habitats in rice fields are better suited to aquatic-type natural enemies, with terrestrial natural enemies generally exhibiting lower survival rates [53]. This study further clarifies the limitations of landscape shrubs in supporting terrestrial natural enemies. The disparity in natural enemy diversity between the two seasons (Table 3) can be explained by the fundamental shift in both pest complexes and habitat characteristics. While the terrestrial wheat system is dominated by aphids, which exhibit rapid population growth and serious damage risk, the rice system is a wetland-type habitat dominated by planthoppers and stem-borers, with distinct pest population dynamics and damage characteristics. Pest population size, growth rate and community structure thus differ fundamentally between wheat and rice systems [51,52]. The semi-aquatic conditions of paddy fields act as an environmental filter, restricting the movement and survival of many terrestrial natural enemies (e.g., ladybirds and spiders). Our findings highlight that during this resource-scarce rice season, the evergreen canopy of *P.* × *fraseri* provides a critical terrestrial refuge, bridging the life-cycle gap for these beneficial insects when the surrounding crop environment becomes unsuitable.

The consistent seasonal trends of dominant natural enemies highlight the synergistic conservation effects of the two plant species: synchronous support for the peak abundance of natural enemy populations during the wheat season, coupled with the two plants providing refuge during the transition and rice seasons to delay population decline. This complementary resource synergy is consistent with prior research suggesting that “combinations of functional plants enhance conservation outcomes” [54]. Previous studies have demonstrated that diverse combinations of functional plants improve the stability of natural enemy communities [55]. The present research provides a concrete case study of landscape shrub combinations, validating prior conclusions that “diverse vegetation configurations enhance pest control functions” [56,57], and further confirming the potential of combining “habitat-type” (*P.* × *fraseri*) and “resource-type” (*P. tobira*) shrubs to stabilize natural enemy populations. This specific configuration compensates for individual plant limitations, such as the depletion of nectar in *P. tobira* post-flowering, thereby extending the duration of natural enemy retention in farmland margins and enhancing the overall potential for biological control [56,57]. From a landscape ecology perspective, *P.* × *fraseri* and *P. tobira* function as linear habitats that act as ecological corridors, facilitating natural enemy migration and providing transitional refuge. Crucially, as both species are evergreen shrubs, they offer persistent structural complexity and thermal buffering throughout the year. Evergreens provide a vital “climatic refuge” and overwintering site for terrestrial natural enemies (e.g., spiders and ladybirds) during the winter fallow or early wheat stages, effectively bridging the critical gap in their life cycles across crop rotations. This aligns with prior findings that “linear non-crop habitats enhance ecological connectivity in farmland” [58,59]. By serving as perennial linear habitats, these evergreen shrub strips act as more than just corridors; they function as year-round thermal and structural refuges. The persistent canopy of *P.* × *fraseri* and *P. tobira* effectively bridges the life-cycle gaps for terrestrial natural enemies, such as ladybirds and spiders, during winter fallow periods. This spatial and temporal connectivity is crucial for maintaining an early-spring population base that can rapidly colonize wheat fields as pest pressure increases. This highlights the indispensable role of permanent, evergreen vegetation in maintaining an initial natural enemy reservoir, which is critical for suppressing pest outbreaks in early spring. Such a consistently maintained natural enemy base ensures sufficient top-down pressure in key periods, which is conducive to keeping pest populations below the economic damage threshold and reducing the risk of pest outbreaks in the rice–wheat rotation system.

Based on this, a deployment strategy is proposed: concentrated, mixed planting along field margins to establish a connected pattern, using a combination of the two abovementioned plant species to compensate for seasonal resource limitations, maintaining appropriate density and low-intensity management to maximize synergistic conservation effects. This configuration is designed to maintain a stable natural enemy reservoir throughout the year; however, the absence of synchronous pest population data remains a limitation of the current study. The primary limitation of the current study is the absence of synchronous pest population data, which restricted our ability to directly quantify the pest suppression efficacy of natural enemy assemblages. Future research may focus on two directions: first, investigating the mechanisms by which differences in plant volatile compounds drive the specific attraction of natural enemies. This direction is aligned with prior research on “plant chemical signals regulating the behavior of natural enemies” [60]. By measuring the volatile components of the two plant species in this study during different phenological stages, detailed insights can be provided to support the precise configuration of vegetation [61,62]. Second, evaluating the dispersal efficiency and pest control efficacy of natural enemies from hedge strips to crop fields [63] would establish a complete “plant–natural enemy–pest” regulatory chain, thereby further validating its ecological control value.

## 5. Conclusions

This study confirms that *P.* × *fraseri* and *P. tobira* are potential synergistic “natural enemy banks” in rice–wheat systems, supporting 31 genera of natural enemies with distinct functional differentiation. *P.* × *fraseri* acts as a habitat-type plant providing stable shelter, while *P. tobira* functions as a resource-type plant supplying nutritional subsidies. Their synergy sustains shared dominant groups like *Harmonia axyridis*, *Cotesia* spp., and *Episyrphus balteatus*, with *P.* × *fraseri* effectively compensating for *P. tobira*’s lower retention capacity during transitional and rice seasons. This study establishes the foundational conservation potential of these landscape plants; however, direct pest suppression outcomes were not measured. When configured as field-edge hedges, these shrubs may facilitate natural enemy dispersal into crop fields, providing a theoretical and practical basis for precise functional plant deployment in ecological pest management (EPM).

## Figures and Tables

**Table 1:** List of dominant predatory natural enemies found on *Photinia* × *fraseri* and their associated host or prey species.

Natural Enemy	Scientific Name	Host/Prey Species
Predatory natural enemy	*Pirata piraticus*	*Nilaparvata lugens*, *Sogatella furcifera*, *Nephotettix virescens*
	*Gnathonarium dentatum*	*Sitobion avenae*, *Rhopalosiphum padi*, *Schizaphis graminum*
	*Clubiona japonicola*	*S. avenae*, *R. padi*, *N. lugens*
	*Episyrphus balteatus*	*S. avenae*, *R. padi*, *S. graminum*
	*Sphaerophoria scripta*	*S. avenae*, *R. padi*, *S. graminum*
	*Melanostoma mellinum*	*S. avenae*, *R. padi*
	*Harmonia axyridis*	*S. avenae*, *R. padi*, *S. graminum*
	*Propylea japonica*	*N. lugens*, *S. furcifera*, *S. avenae*, *Aphis gossypii*
Parasitic natural enemy	*Cotesia* spp.	*Plutella xylostella*, *Pieris rapae*, *Mythimna separata*
	*Microplitis tuberculifer*	*Agrotis tokionis*, *Agrotis segetum*, *M. separata*
	*Opius* sp.	*Hydrellia chinensis*, *Meromyza saltatrix*
	*Opiognathus aulaciferus*	*Agromyzidae* (e.g., *Liriomyza* spp.)

**Table 2:** List of dominant parasitic natural enemies found on *Pittosporum tobira* and their associated host or prey species.

	Scientific Name	Host/Prey Species
Predatory natural enemy	*Pirata piraticus*	*Nilaparvata lugens*, *Sogatella furcifera*, *Nephotettix virescens*
	*Gnathonarium dentatum*	*Sitobion avenae*, *Rhopalosiphum padi*, *Schizaphis graminum*
	*Clubiona japonicola*	*S. avenae*, *R. padi*, *N. lugens*
	*Episyrphus balteatus*	*S. avenae*, *R. padi*, *S. graminum*
	*Sphaerophoria scripta*	*S. avenae*, *R. padi*, *S. graminum*
	*Sphaerophoria menthastri*	Aphids (e.g., *S. avenae*)
	*Harmonia axyridis*	*S. avenae*, *R. padi*, *S. graminum*
Parasitic natural enemy	*Cotesia* spp.	*Plutella xylostella*, *Pieris rapae*, *Mythimna separata*
	*Microplitis tuberculifer*	*Agrotis tokionis*, *Agrotis segetum*, *M. separata*
	*Dolichogenidea baoris*	*Parnara guttata*, *Pelopidas mathias*
	*Opius* sp.	*Hydrellia chinensis*, *Meromyza saltatrix*
	*Anagrus* sp.	*N. lugens*, *S. furcifera*, *N. virescens*
	*Opiognathus aulaciferus*	*Agromyzidae* (e.g., *Liriomyza* spp.)

**Table 3:** Alpha diversity indices of natural enemy communities associated with *Photinia* × *fraseri* and across different planting seasons.

Landscape Plants	Different Planting Seasons	ASV	Shannon	Simpson	Chao1	ACE
*Photinia* × *fraseri*	Wheat	10.00 ± 0.56 a	1.31 ± 0.04 ab	0.63 ± 0.02 b	10.08 ± 0.61 a	10.43 ± 0.69 a
	Wheat–Rice	13.00 ± 0.78 b	1.80 ± 0.06 c	0.77 ± 0.01 c	15.50 ± 1.56 b	14.95 ± 1.09 b
	Rice	6.87 ± 0.63 c	1.27 ± 0.06 a	0.63 ± 0.03 b	7.75 ± 1.43 c	8.21 ± 0.97 c
*Pittosporum tobira*	Wheat	8.00 ± 0.37 a	1.58 ± 0.02 a	0.74 ± 0.01 a	8.33 ± 0.42 a	8.67 ± 0.61 a
	Wheat–Rice	11.67 ± 0.76 b	1.95 ± 0.04 b	0.80 ± 0.01 b	16.83 ± 3.65 b	15.43 ± 2.11 b
	Rice	8.81 ± 0.61 c	1.32 ± 0.09 c	0.64 ± 0.04 c	9.06 ± 0.78 c	9.78 ± 0.89 c

Note: Data are expressed as mean ± SE. Observed ASVs represent the number of detected taxonomic units. Different lowercase letters within the same column for each location indicate significant differences between seasons at *p* < 0.05 (one-way ANOVA with Duncan’s multiple range test).

## Data Availability

The original contributions presented in this study are included in the article/Supplementary Materials. Further inquiries can be directed to the corresponding authors.

## Supplementary Materials

The following supporting information can be downloaded at https://www.mdpi.com/article/10.3390/insects17040428/s1. Figure S1: Schematic diagram of the experimental plot design, showing the arrangement of the 80 m long *Photinia* × *fraseri* hedgerow and the randomized placement of three Malaise traps (indicated by black triangles); Figure S2: Schematic diagram of the experimental plot design, showing the arrangement of the 80 m long *Pittosporum tobira* landscape belt and the randomized placement of three Malaise traps (indicated by black triangles); Table S1: Detailed sampling schedule for natural enemies in *Photinia* × *fraseri* and *Pittosporum tobira* treatments across the wheat season, wheat–rice transition, and rice season.
