# Supplementary material for: The Potential of Landscape Plants Photinia × fraseri and Pittosporum tobira as Refuge for Natural Enemies of Pest Insects in Rice–Wheat Rotation Systems
Source: Insects. 2026 Apr 16;17(4):428. doi: 10.3390/insects17040428 (PMC13116421; doi:10.3390/insects17040428)
Supplement: Supplementary file 1 [file insects-17-00428-s001.zip › insects-4202796-supplementary.pdf]

Table S1: Detailed sampling schedule for natural enemies in *Photinia × fraseri* and *Pittosporum tobira* treatments across the wheat season, wheat–rice transition, and rice season.

Sampling area	Agricultural landscape plants	Sampling date		
		Wheat Season	Wheat-Rice Transition Season	Rice Season
Jiashan	*Pittosporum tobira*	2024.05.02	2024.06.18	2024.08.16
		2024.05.16	2024.07.04	2024.08.30
			2024.07.15	2024.09.12
			2024.08.01	2024.09.26
				2024.10.11
				2024.11.07
Deqing	*Photinia × fraseri*	2024.04.29	2024.05.29	2024.08.29
		2024.05.13	2024.07.16	2024.09.11
				2024.09.26
				2024.10.10
				2024.11.07

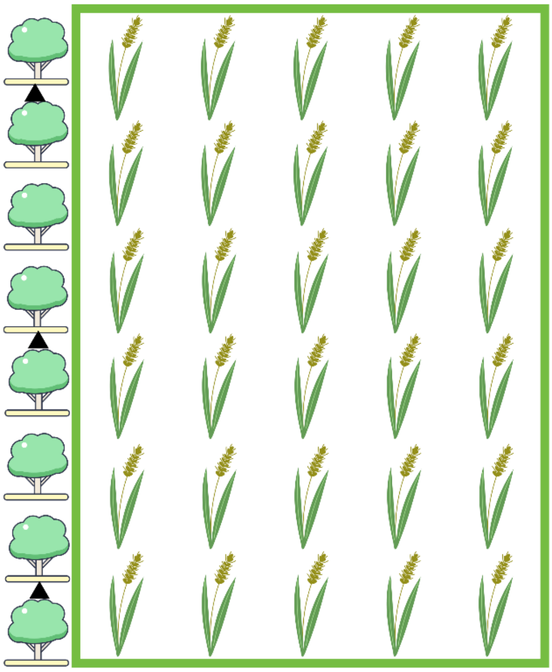

Figure S1: Schematic diagram of the experimental plot design, showing the arrangement of the 80 m long *Photinia* × *fraseri* hedgerow and the randomized placement of three Malaise traps (indicated by black triangles).

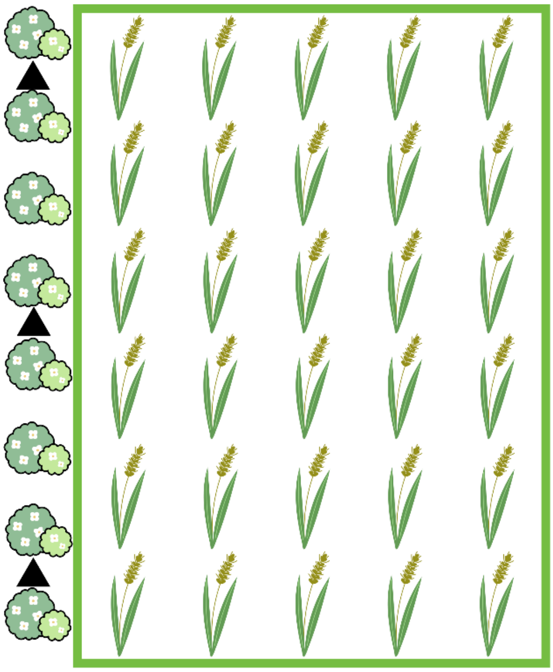

Figure S2: Schematic diagram of the experimental plot design, showing the arrangement of the 80 m long *Pittosporum tobira* landscape belt and the randomized placement of three Malaise traps (indicated by black triangles).
